# *findMySequence*: a neural-network-based approach for identification of unknown proteins in X-ray crystallography and cryo-EM

**DOI:** 10.1107/S2052252521011088

**Published:** 2021-12-01

**Authors:** Grzegorz Chojnowski, Adam J. Simpkin, Diego A. Leonardo, Wolfram Seifert-Davila, Dan E. Vivas-Ruiz, Ronan M. Keegan, Daniel J. Rigden

**Affiliations:** a European Molecular Biology Laboratory, Hamburg Unit, Notkestrasse 85, 22607 Hamburg, Germany; b Institute of Systems, Molecular and Integrative Biology, University of Liverpool, Liverpool L69 7ZB, United Kingdom; c São Carlos Institute of Physics, University of São Paulo, Avenida João Dagnone 1100, São Carlos, SP 13563-120, Brazil; d European Molecular Biology Laboratory, Meyerhofstraße 1, 69117 Heidelberg, Germany; e Laboratorio de Biología Molecular, Facultad de Ciencias Biológicas, Universidad Nacional Mayor de San Marcos, Avenida Venezuela Cdra 34 S/N, Ciudad Universitaria, Lima, Peru; f Rutherford Appleton Laboratory, Research Complex at Harwell, UKRI-STFC, Didcot OX11 0FA, United Kingdom

**Keywords:** protein structures, protein sequences, *SIMBAD*, cryo-EM, bioinformatics, structure determination, *findMySequence*, neural networks

## Abstract

Although experimental protein-structure determination usually targets known proteins, chains of unknown sequence are often encountered. They can be purified from natural sources, appear as an unexpected fragment of a well characterized protein or appear as a contaminant. Regardless of the source of the problem, the unknown protein always requires characterization. Here, an automated pipeline is presented for the identification of protein sequences from cryo-EM reconstructions and crystallographic data. The method’s application to characterize the crystal structure of an unknown protein purified from a snake venom is presented. It is also shown that the approach can be successfully applied to the identification of protein sequences and validation of sequence assignments in cryo-EM protein structures.

## Abstract 2

*findMySequence* is presented – a machine-learning method for the identification of unknown proteins and sequence-assignment validation in cryo-EM and X-ray crystallography.

## 1. Introduction

Recent years have witnessed an unprecedented advancement of protein-structure-prediction approaches. Tools based on deep neural networks proved not only much better than any previously available approaches but also able to predict structures of proteins at a level of detail comparable with X-ray crystallography, which has been traditionally the predominant high-resolution technique (Jumper *et al.*, 2021 ▸). Nevertheless, there are macromolecular targets which are not yet amenable to *in silico* structure-prediction approaches, most notably including structurally heterogeneous large macromolecular complexes containing protein, RNA and small-molecule components. Particularly interesting in this context are recent advances in cryo-EM that enabled detailed studies of macromolecular complexes in their natural cellular environment (Tegunov *et al.*, 2021 ▸). The biochemical factors affecting the macromolecular composition and conformation are, however, not the only issue here as whole macromolecular complexes or their components may remain uncharacterized prior to structure-determination attempts, or when purified from endogenous sources (Roh *et al.*, 2018 ▸). The problem of unknown-protein identity is not unique to cryo-EM studies. It is surprisingly common for macromolecular crystallographers to crystallize and solve previously uncharacterized protein structures. These can be proteins purified from natural sources (as described in this work) or contaminants, either native to an expression host (Niedzialkowska *et al.*, 2016 ▸) or from elsewhere (Keegan *et al.*, 2016 ▸; Hatti *et al.*, 2016 ▸). Finally, samples can be simply mislabeled during crystallization or a synchrotron trip, which we have witnessed surprisingly often.

The experimental method of choice for protein characterization is sequencing of a sample (preparation or a crystal) using mass spectrometry. In the case of cryo-EM, however, this may reduce the number of plausible sequence alternatives but not solve the sequence-assignment problem entirely (Ramrath *et al.*, 2018 ▸). It is also not applicable to crystallography in cases where a crystal and sample solution are no longer available and cannot be reproduced.

Solving the crystal structure of a mis- or unidentified protein is a major difficulty. When reliable phase estimates can be obtained from anomalous differences, or directly from ultra-high-resolution diffraction data, an initial model can be built based on an experimentally phased electron-density map. The use of a standard alternative-phasing method – molecular replacement (MR) – is typically impossible due to lack of model-selection criteria. In such a case, a brute-force MR approach, such as that implemented in *SIMBAD* (Simpkin *et al.*, 2020 ▸, 2018 ▸), *marathonMR* (Hatti *et al.*, 2017 ▸) or by the *Wide-Search MR* server (Stokes-Rees & Sliz, 2010 ▸), is the only option.

In a high-resolution crystal structure model the manual identification of protein sequences can be performed by an experienced crystallographer. However, similarly shaped amino acid side chains (*e.g.* glutamate and glutamine, or aspartate and asparagine) cannot usually be distinguished without an additional source of information. At lower resolutions the procedure becomes increasingly difficult, as model tracing itself is non-trivial without the sequence information (Chojnowski *et al.*, 2019 ▸). In such cases, when at least a fragmented model can be traced in an electron-density map, the use of fold-recognition tools [such as *GESAMT* (Krissinel, 2012 ▸), *DALI* (Holm & Laakso, 2016 ▸) or *FATCAT* (Ye & Godzik, 2003 ▸)] may help to identify the protein (Niedzialkowska *et al.*, 2016 ▸). This approach, however, may be difficult as the identification of complete protein chains in fragmented models, and in the presence of symmetry, is usually not possible without the use of a target sequence. On the other hand, the use of short polypeptide stretches may be misleading as many remote homologs tend to share structural motifs (Chojnowski *et al.*, 2020 ▸).

For cryo-EM, the sequence-identification methods mentioned above in the context of X-ray crystallography are generally applicable. Nevertheless, cryo-EM maps are usually determined at resolutions lower than in crystallography, making the model building and the identification of side-chain identities far more ambiguous and challenging (Chojnowski *et al.*, 2021 ▸). There are, however, examples of successful protein identification that avoid these issues by a brute-force fitting of structures automatically predicted for a large pool of proteome sequences (Ramrath *et al.*, 2018 ▸). It was also shown (Brown *et al.*, 2015 ▸) that a large-scale rigid-body docking of protein domains using a *BALBES*–*MOLREP* pipeline (Long *et al.*, 2008 ▸; Vagin & Teplyakov, 1997 ▸) may enable successful identification of target protein homologues from the Protein Data Bank (PDB) (Berman *et al.*, 2000 ▸).

Several computer programs have previously been developed to facilitate protein identification in X-ray crystallography and cryo-EM. *Fitmunk* (Porebski *et al.*, 2016 ▸), originally side-chain modelling software, can assign probable residue identities to partial crystal structure models to then query sequence databases using *BLAST* (Altschul *et al.*, 1997 ▸). Although this approach generously assumes that the residue-type ambiguity can be modelled using standard scoring matrices hardcoded in *BLAST*, it has been successfully used for the determination of sequence identity in several protein models (Niedzialkowska *et al.*, 2016 ▸). The program *phenix.sequence_from_map* (Terwilliger, 2003 ▸) estimates residue-type probabilities based on the correlation of side-chain rotamer templates with a map, which can then be used to query sequence databases. *CryoID* (Ho *et al.*, 2020 ▸), an approach designed to address protein characterization in cryo-EM, uses the *phenix.sequence_from_map* tool adapted for the interpretation of EM maps to identify plausible residue identities using a six-letter sequence, which is simplified based on side-chain volume similarity. Similarly to *Fitmunk*, *CryoID* relies on standard sequence-similarity scoring matrices that have been adapted for the simplified six-letter sequence. It also requires manual curation and selection of the most reliable fragments, presumably due to the fact that by default the method strongly penalizes gaps in *BLAST* alignments and requires reliable continuous main-chain traces on input. In a recent update (published when our article was in preparation) *CryoID* authors modified their protocol, which instead of *BLAST* can also use a dynamic programming sequence-alignment procedure that accounts for tracing errors in the models (Terwilliger *et al.*, 2021 ▸). Although the alignments are obtained by default using a simplified six-letter sequence, they are scored using individual residue probabilities derived from a rotamer-template-matching procedure implemented in *phenix.sequence_from_map*.

It is known that tracing errors (deletions, insertions) are very common in intermediate models, in particular at lower resolutions and cryo-EM models, and correcting them usually requires the use of a target sequence (Chojnowski *et al.*, 2019 ▸). This has already been recognized by the authors of the above-mentioned *CryoID* update. Moreover, standard substitution matrices used for sequence alignment do not necessarily reflect the ambiguity of the residue types assigned based on partial models and maps. For example, in a recent study it was shown that a machine-learning classifier most reliably discriminated shortest side chains (alanine versus glycine) that would fall into a single group simplified by side-chain size only (Chojnowski *et al.*, 2019 ▸). To address these issues we developed *findMy­Sequence*, a computer program that uses machine-learning predicted residue-type probabilities to query sequence databases using *HMMER*, a popular sequence-analysis tool (Eddy, 2011 ▸). We show that the program successfully identifies sequences of protein models automatically built into crystallographic and cryo-EM maps, even though the automatically built models are usually highly fragmented and prone to tracing errors (Chojnowski *et al.*, 2021 ▸). Furthermore, we use *findMy­Sequence* to identify a protein purified and crystallized from a snake venom retrieved from *Bothrops atrox*, the most clinically important snake species in northern parts of South America (Estevao-Costa *et al.*, 2016 ▸).

## 2. Materials and methods

### 2.1. Crystal structures training set

For training the crystal structure residue-type classifier, we selected protein crystal structures from the PDB (Berman *et al.*, 2000 ▸). The selection criteria included pairwise sequence identity below 50% (PDB50 set), resolution between 2 and 3 Å, and crystallographic *R* factor below 0.3. Out of 54 749 structures fulfilling these criteria on 4 February 2020, we selected 1000 at random and downloaded corresponding ‘conservatively optimized’ crystal structure models from the *PDB_REDO* server (Joosten *et al.*, 2014 ▸) together with the corresponding weighted 2*F*_o_ − *F*_c_ map coefficients from *REFMAC5* (Murshudov *et al.*, 2011 ▸).

### 2.2. EM-structures training set

For training the cryo-EM residue-type classifier, we selected from PDB cryo-EM structures solved at a resolution better than 4 Å, with molecular weight below 500 kDa and half maps available for download in the Electron Microscopy Data Bank (EMDB) (Velankar *et al.*, 2016 ▸). As of 4 February 2020, we found 184 structures fulfilling these criteria. Initially, all the structures were refined into their corresponding maps with *REFMAC5* using the auto_em.sh script from the *ARP/wARP* 8.1 suite (Chojnowski *et al.*, 2021 ▸). Out of these, 117 models with CC_mask over 0.7 [estimated using *phenix.map_model_cc* (Liebschner *et al.*, 2019 ▸)] were selected for training the classifier. The deposited maps were not altered by any means prior to training.

### 2.3. Crystal structure identification benchmark set

For benchmarking the sequence-identification procedure in crystal structures, we used a set of main-chain-only models built using *ARP/wARP* for MR solutions at various target resolutions and search-model similarity levels.

As targets we selected three hen egg-white lysozyme (HEWL) crystal structures solved over a range of resolutions: 2.9, 2.2 and 1.2 Å (PDB IDs 4gce, 4rln and 2hub, respectively) (Helliwell & Tanley, 2013 ▸; Botha *et al.*, 2015 ▸; Lagziel-Simis *et al.*, to be published). All the target structures contain one molecule and 129 residues in the asymmetric unit (ASU) and were solved in space group *P*4_3_2_1_2, albeit with slightly different unit-cell dimensions.

For each of the targets we used *GESAMT* (Krissinel, 2012 ▸) with default parameters to select a set of structurally similar models from the PDB (as of 4 February 2020). After excluding structures with ‘CA atoms only’ and those solved with powder diffraction or NMR, the sets contained 1496, 1478 and 1492 models for targets at 2.9, 2.2 and 1.2 Å resolution, respectively, spanning over large ranges of sequence identity and structural similarity to the target (see Fig. S1 in the supporting information) The different numbers of search models can be attributed to small differences in the target-structure coordinates. We used these as search models to solve the corresponding target structure with MR using *Phaser* (McCoy *et al.*, 2007 ▸) with default parameters. The MR solutions were then used as an input for main-chain-only model building with *ARP/wARP* 8.1. In addition to default model-building parameters, we employed a recently developed protocol for building short loops without sequence information (Chojnowski *et al.*, 2019 ▸) to reduce model fragmentation. For benchmarks we used final *ARP/wARP* models and corresponding weighted 2*F*_o_ − *F*_c_ maps from *REFMAC5*.

### 2.4. EM-structure-identification benchmark set

To benchmark the sequence-identification procedure in cryo-EM maps, we used two sets of ribosomal proteins: models built *de novo* and deposited models refined into corresponding EM maps.

From the PDB we selected cryo-EM structures of ribosomes determined at a resolution better than 3.5 Å, with half maps available for download in the EMDB. Initially, all the structures were refined into corresponding maps with *REFMAC5* using the auto_em.sh script from *ARP/wARP* 8.1 suite (Chojnowski *et al.*, 2021 ▸). Out of these we selected refined models with CC_mask over 0.7 [estimated using *phenix.map_model_cc* (Liebschner *et al.*, 2019 ▸)]. The resulting set contained 17 ribosomes and 909 protein-chain models originating from five different organisms: *Plasmodium falciparum, Escherichia coli, Staphylococcus aureus, Sus scrofa* and *Oryctolagus cuniculus*.

For each of the protein-chain models we estimated median local resolution for CA atom positions, using local resolution maps calculated using *ResMap* version 1.1.4 (Kucukelbir *et al.*, 2014 ▸) with default parameters.

For benchmarks we used both deposited coordinates of ribosomal proteins and corresponding main-chain models traced fully automatically and *de novo* using *ARP/wARP* 8.1 with default parameters. To reduce model fragmentation we additionally used a recently developed protocol for building short loops without sequence information (Chojnowski *et al.*, 2019 ▸). Each protein-chain model was built in an artificial rectangular box encapsulating a corresponding deposited model with a 5 Å margin. All remaining protein and RNA atoms from the corresponding ribosome model were masked with 3.0 Å radius.

### 2.5. Sequence databases

In principle, the protein-sequence-identification queries can be carried out against any set of sequences in FASTA format. Here, we used a set of 552 121 sequences corresponding to all protein chains available in the PDB, downloaded as of 17 September 2020 (PDB100). For the identification of ribosomal proteins in cryo-EM models we also used sets of reference proteomes downloaded from UniProt (The UniProt Consortium, 2021 ▸) for each of the targets. These were significantly smaller than the PDB100 set: three contained less than 6000 sequences (*P. falciparum, E. coli, S. aureus*; UniProt IDs UP000001450, UP000000625 and UP000008816, respectively), while two mammalian proteomes had roughly 20 000 sequences each (*S. scrofa, O. cuniculus*; UniProt IDs UP000008227 and UP000001811, respectively).

*Saccharomyces cerevisiae* proteome used for the identification of Voa1 assembly factor was downloaded from UniProt (UP000002311, 6049 sequences). *B. atrox* proteome sequences identified by Amazonas *et al.* (2018 ▸) were downloaded from UniProt (selected by taxonomic identifier 8725).

### 2.6. Solving crystal structures with *SIMBAD*

**Figure 1:**
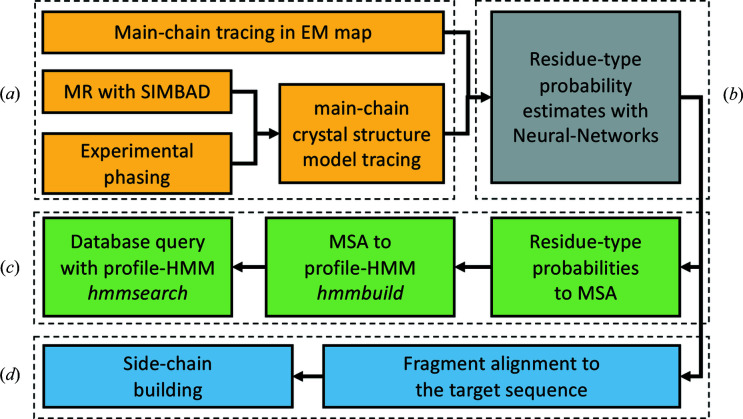
A schematic representation of the *findMy­Sequence* usage workflow. Key steps are grouped in dashed rectangles: (*a*) structure solution and model building, (*b*) model interpretation, (*c*) sequence-database queries, and (*d*) sequence assignment and model building. All steps except model tracing (*a*) are integrated in the software and performed automatically.

In contrast to cryo-EM, where a complete map can be reconstructed from experimental data, the phases in crystallography are not measured and need to be retrieved from other sources [Fig. 1 ▸(*a*)]. MR exploits the fact that evolutionarily related macromolecules tend to be structurally similar. Given sufficient similarity, a known structure correctly positioned in the target cell by MR can provide an approximation to the unknown phases of the target. In a typical MR search, suitable search models can be identified by a sequence search. However, in the case of the venom protein crystal discussed here, the target sequence was unknown. Therefore the domain-database search option in *SIMBAD* was used to perform sequence-independent MR. This brute-force option makes use of the non-redundant domain database defined in the *MoRDa* application (Vagin & Lebedev, 2015 ▸) consisting of almost 100 000 domains from the PDB. These domains are used as search models in the rotation function step of MR as a quick means to score and identify possible homologues suited to providing the initial approximation to the target’s phases.

### 2.7. Neural-network-model architecture and training

For predicting residue-type probabilities based on map values and main-chain models we built two neural-network models [Fig. 1 ▸(*b*)]. The two models have identical architecture but are trained on distinct training sets derived from crystal structures or cryo-EM models and their respective maps (see Sections 2.1 and 2.2 for details).

Side-chain densities are described as a vector containing 324 map values sampled on a regular grid with 1.0 Å spacing (a residue descriptor). The grid is defined by N-, CA- and C-peptide-backbone atoms; it is centred at the CA atom and spanned by orthonormal vectors defined by N–CA atoms (**e_x_**), N–CA–C plane normal (**e_y_**) and their cross product (**e_z_** = **e_x_** × **e_y_**). The input to the classifier contains all grid points that are within 1.0 Å distance from any side-chain atom in the top500 rotamers library (Lovell *et al.*, 2000 ▸) aligned by N-, CA- and C-backbone atoms. The alignment of the classifier input (residue descriptor) to the main-chain atoms makes it very sensitive to tracing errors, which is a desired feature of the method.

The neural-network-model input is a vector of length 324 (the residue descriptor). The model contains two fully connected hidden layers. The first layer has a rectified-linear-unit activation function, which sets all negative neuron inputs to zero, and 324 output features. The second layer has 20 output features and uses the log-softmax normalization function, enabling estimation of output classification probabilities. To avoid overfitting, we inserted an additional dropout layer between the two hidden layers, which at each training step disables neuron connections at random with probability *p* = 0.5. The models were trained for 1000 epochs with a batch size of 20 residue descriptors in each parameters update cycle and a 10% validation set. The models were trained using the Adam optimization algorithm (Kingma & Ba, 2014 ▸) with a learning rate of 1 × 10^−4^ that resulted in the best test-set accuracies. For training the crystal structure classifier, we used 617 477 and 68 608 residue descriptors for training and test set, respectively. The accuracies of a resulting model were 0.88 for the training set and 0.86 for the test set.

Similarly, for training the EM classifier we used 177 831 and 19 758 residue descriptors in training and test sets that resulted in a model with estimated accuracies of 0.64 and 0.59 for training and test sets, respectively. We observed that unlike a support-vector machine-based classifier trained previously for the sequence assignment of crystal structure models in *ARP/wARP* (Chojnowski *et al.*, 2019 ▸), the accuracy of the neural network only weakly depends on residue type. This, combined with the overall high accuracy of the classifier, makes it a substantial improvement on the previously published method, which results in a clearly improved performance of the sequence-identification procedure (Fig. S2).

### 2.8. Making queries in sequence databases with *HMMER*

To find a sequence in a database that matches the predicted residue-type probabilities, we use sequence-comparison tools from the *HMMER* suite (Eddy, 2011 ▸) [Fig. 1 ▸(*c*)]. Initially, predicted residue-type probabilities are converted into a multiple sequence alignment (MSA), where fractions of residue types in each column correspond to predicted probabilities. The residues in the input model are processed sequentially, starting from the longest continuous chain fragments. Next, the MSA is converted into a profile hidden Markov model (profile-HMM) using the default configuration of the *hmmbuild* program from the *HMMER* suite. Finally, the profile-HMM is used to query a sequence database using *hmmsearch* with default parameters, and sequences with the lowest best-single-domain *E* values (3 by default) are returned to the user.

### 2.9. Sequence assignment in main-chain models

To build side chains in the input main-chain model fragment [Fig. 1 ▸(*c*)], we consider all possible alignments of a fragment to the target sequence. The most plausible alignment, given predicted residue-type probabilities, is then used to assign residue types to the fragment. For computational efficiency, we only consider alignments of the fragments as a whole, which ignores tracing errors (insertions, deletions or wrong connections).

Individual alignments are scored with a sum of log probabilities of finding a specific residue type at consecutive positions in a chain fragment. Although our machine-learning classifier has been calibrated and the predicted residue-type probabilities generally reflect expected frequencies, the accuracy of predictions may vary depending on resolution and quality of the models (Chojnowski *et al.*, 2019 ▸). Therefore, for each alignment we calculate a standard score (*Z* score) using log-probability distribution parameters estimated for a given fragment and a random target sequence. To additionally account for the varying target-sequence length we estimate a probability that the highest *Z* score selected from a number of alternative alignments of a fragment to the target sequence was observed by chance (*p* value). For this purpose we apply Gumbel–Fisher–Tippett extreme-value distribution theorem using formulas derived previously for normalized structure-factor amplitudes (Chojnowski & Bochtler, 2007 ▸). The analysis assumes that the *Z* scores are normally distributed, which we confirmed on our benchmark set using the Shapiro–Wilk test at 99% confidence level (Shapiro & Wilk, 1965 ▸).

The extreme-value analysis allows for the comparison of scores obtained for multiple target sequences of various lengths. This, however, assumes that all the alternative alignments of a chain fragment to the target sequence are statistically independent, which is obviously not the case. To account for that, for *p*-value estimates we use a reduced number of alternative fragment alignments to the target sequence. According to our systematic analysis, reducing this number by a factor of ten results in *p* values closest to the logistic regression estimates of the correct assignment probabilities in our benchmark set.

### 2.10. Venom protein purification, crystallization and data processing

A manual venom extraction on the main venom gland of *B. atrox* from Pucallpa (Peru) was carried out. The venom was lyophilized and stored at −10°C until later use. A total of 350 mg of lyophilized venom from *B. atrox* was dissolved in 10 ml of 50 m*M* ammonium acetate buffer pH 5.0. The resuspended venom was centrifuged at 2000*g* for 20 min at room temperature. The pellet containing insoluble particles was discarded. The clear supernatant was applied to a CM Sephadex ion-exchange column C-50 (28 × 2.6 cm) previously equilibrated with the same buffer and the isocratic elution of unbound proteins was monitored at 280 nm. Bound proteins were eluted with a gradient (0–1 *M* NaCl) at a flow rate of 1 ml min^−1^. In order to identify hemolytically active components, all eluted fractions were evaluated with the indirect hemolytic assay described previously (Camey *et al.*, 2002 ▸). The fractions with the greatest activity, corresponding to the same peak, were pooled and concentrated for size-exclusion purification. The concentrated fraction was applied to a Superdex 75 10/300 GL column, previously equilibrated with 50 m*M* ammonium acetate buffer pH 5.0. The enzymatic activity was monitored, and the purity of the protein was evaluated with SDS–PAGE.

The purified protein with hemolytic activity was concentrated to 11 mg ml^−1^ in 50 m*M* ammonium acetate buffer pH 5.0. Crystallization screening was performed with the sitting-drop vapour-diffusion method in 96-well plates using a Honeybee 931 robot (Genomic Solutions Inc.) and a commercially available Crystal Screen II kit at 18°C. After seven days, single crystals [20%(*v*/*v*) 2-propanol, 20%(*w*/*v*) PEG 4000 and 0.1 *M* sodium citrate] were harvested and cryo-cooled in liquid nitro­gen for data collection. X-ray diffraction data were collected on beamline MX-2 at the synchrotron-radiation source at the Brazilian National Laboratory of Synchrotron Light from the National Center for Energy and Materials (LNLS–CNPEM, Brazil) housing a PILATUS 2M detector. The data were indexed and integrated in *iMosflm* version 7.2.1 (Battye *et al.*, 2011 ▸), and scaled with *SCALA* (Evans, 2006 ▸) to 1.95 Å resolution (Table 2).

### 2.11. Implementation and availability

The sequence-identification program *findMy­Sequence* was implemented using Python 3 with an extensive use of *PyTorch* (Paszke *et al.*, 2019 ▸), *NumPy* (Oliphant, 2006 ▸), *SciPy* (Virtanen *et al.*, 2020 ▸), *CCTBX* (Grosse-Kunstleve *et al.*, 2002 ▸), and *CCP*4 (Winn *et al.*, 2011 ▸) libraries and utility programs. For making sequence-database queries we used *HMMER* suite version 3.3.2. The program source code and installation instructions are available at https://gitlab.com/gchojnowski/findmysequence.

The model of the snake-venom protein was deposited in the PDB.

## 3. Results

### 3.1. Benchmarks with deposited crystal structure models

To estimate the performance of our sequence-identification procedure we used a large set of main-chain-only protein crystal structure models built using *ARP/wARP* from MR solutions, as described in Section 2.3.

**Figure 2:**
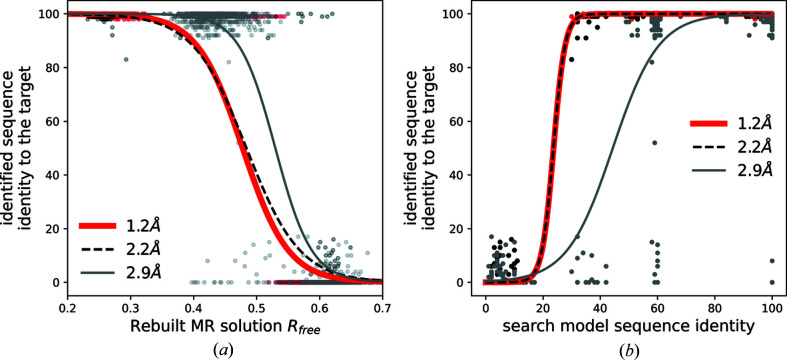
Sequence-identification benchmarks for crystal structure models solved with MR using *Phaser*. Sequence identity of an identified sequence to the target sequence as a function of (*a*) *R*_free_-factor value of a MR solution rebuilt using *ARP/wARP* without an input sequence and (*b*) sequence identity of a MR search model to the target structure. The continuous and dashed curves are logistic regression estimates of a probability that an identified sequence will have at least 80% sequence identity to the target sequence.

We observed that the quality and completeness of a model are main determinants of the success of the procedure. In cases where a reasonable model can be built (*R*_free_ below 50%) a largely correct sequence (above 80% of target residues are correctly assigned) can be identified in the vast majority of cases [Fig. 2 ▸(*a*)]. Although this simple rule of thumb applies to all the tested targets, the performance of model building, and consequently the sequence identification, is clearly reduced at lower resolution (2.9 Å). This is in line with previous observations on the resolution dependence of the *ARP/wARP* model building results (Chojnowski *et al.*, 2020 ▸).

The *R*_free_ values observed here are higher than one would expect from a good quality model. This is due to the presence in the models of ‘free atoms’ used by *ARP/wARP* for the sparse representation of electron-density maps. The atoms are not removed from final models built without sequence information (or with a low sequence coverage). As a consequence, complete main-chain-only models built at lower resolutions are usually moderately overfitted, which results in high *R*_free_ values.

For all three targets, a search model with at least 20% sequence identity is required to solve the structure and identify the corresponding sequence [Fig. 2 ▸(*b*)]. However, for the lowest-resolution dataset, sequence-identification attempts with higher sequence-identity search models were also occasionally unsuccessful.

### 3.2. Benchmarks with cryo-EM ribosomal protein models

**Figure 3:**
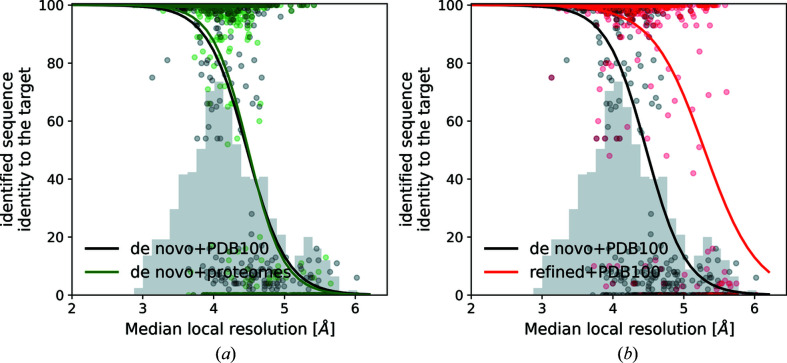
Sequence-identification benchmarks for 909 cryo-EM models of ribosomal proteins. (*a*) Comparison of the method performances for an identification of models built *de novo* against small (proteomes) and large (PDB100) sequence databases. (*b*) Comparison of the method performances for models built *de novo* and those based on refined deposited coordinates. Histograms of the median local resolution of the test-set proteins are shown in grey (in arbitrary units). The continuous curves are logistic regression estimates of a probability that an identified sequence will have at least 80% sequence identity to the target sequence.

We benchmarked our automated sequence-identification procedure on a set of 909 ribosomal protein structures described in Section 2.4. We observed that the majority of sequences could be correctly identified using models built *de novo* up to 4.5 Å local resolution [Fig. 3 ▸(*a*)], which is usually too low for an automated method to trace a complete model (Lawson *et al.*, 2021 ▸). The use of complete deposited models readily increases sequence-identification performance at lower local resolutions [Fig. 3 ▸(*b*)]. We also observed that the sequence-identification performance for models built *de novo* does not depend on the size of a target-sequence database [Fig. 3 ▸(*a*)].

**Figure 4:**
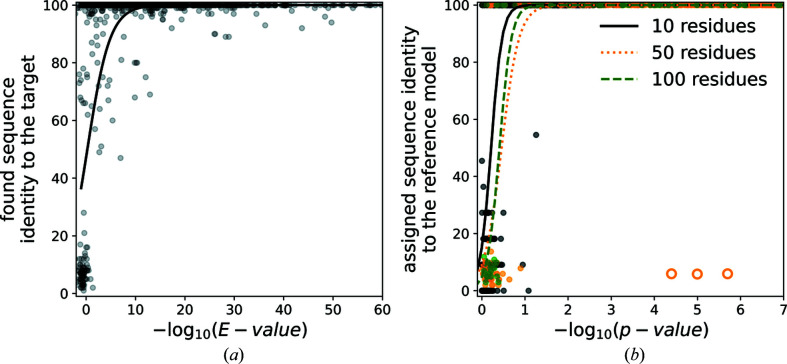
Sequence-identification and assignment benchmarks for EM models. (*a*) Identity of a sequence identified for models built *de novo* using *ARP/wARP* as a function of *HMMsearch* best-single-domain sequence-alignment score. (*b*) Identity of a sequence assigned to continuous fragments of deposited EM models as a function of the sequence-assignment score (*p* value) for protein-fragment lengths of 10, 50 and 100 residues selected at random from test-set models. The continuous curves on the plots are logistic regression estimates of a probability that an identified sequence will have at least 80% sequence identity to the reference model. The orange circles represent three reference chains with register error that were not used for the logistic regression calculations.

We also evaluated the reliability of the best-single-domain *E* value reported by *HMMsearch* [Fig. 4 ▸(*a*)]. Generally, for *E* values below 1 × 10^−7^, a sequence close to the target was identified in most of the cases (the logistic regression estimate of the correct identification probability exceeds 95%). At the same time, however, we observed that a number of hits with very low *E* values do not exactly match the reference, suggesting a limited, albeit very high, accuracy in the approach. In the case of crystal structure models, the logistic regression estimate of the correct identification probability exceeds 95% for *E* values below 1 × 10^−3^. This is in line with our estimates of residue-type classifier accuracy, which is significantly higher for crystal than cryo-EM models.

### 3.3. Benchmarks of the sequence assignment in EM models

We validated the sequence-assignment procedure using the complete test set of ribosomal proteins. From the protein chain in the test set we selected random continuous fragments of length 10, 50 and 100 residues. This resulted in 899, 820 and 548 fragments of length 10, 50 and 100 residues, respectively. Next, we used our sequence-assignment procedure to find an optimal alignment of the fragments to their deposited sequences given the corresponding map.

We observed that the *p* value is a very accurate estimate of sequence-assignment reliability and, regardless of the model quality, local resolution, fragment and target-sequence lengths for *p* values below 1 × 10^−1^, the sequence assignment is unambiguous [Fig. 4 ▸(*b*)].

**Figure 5:**
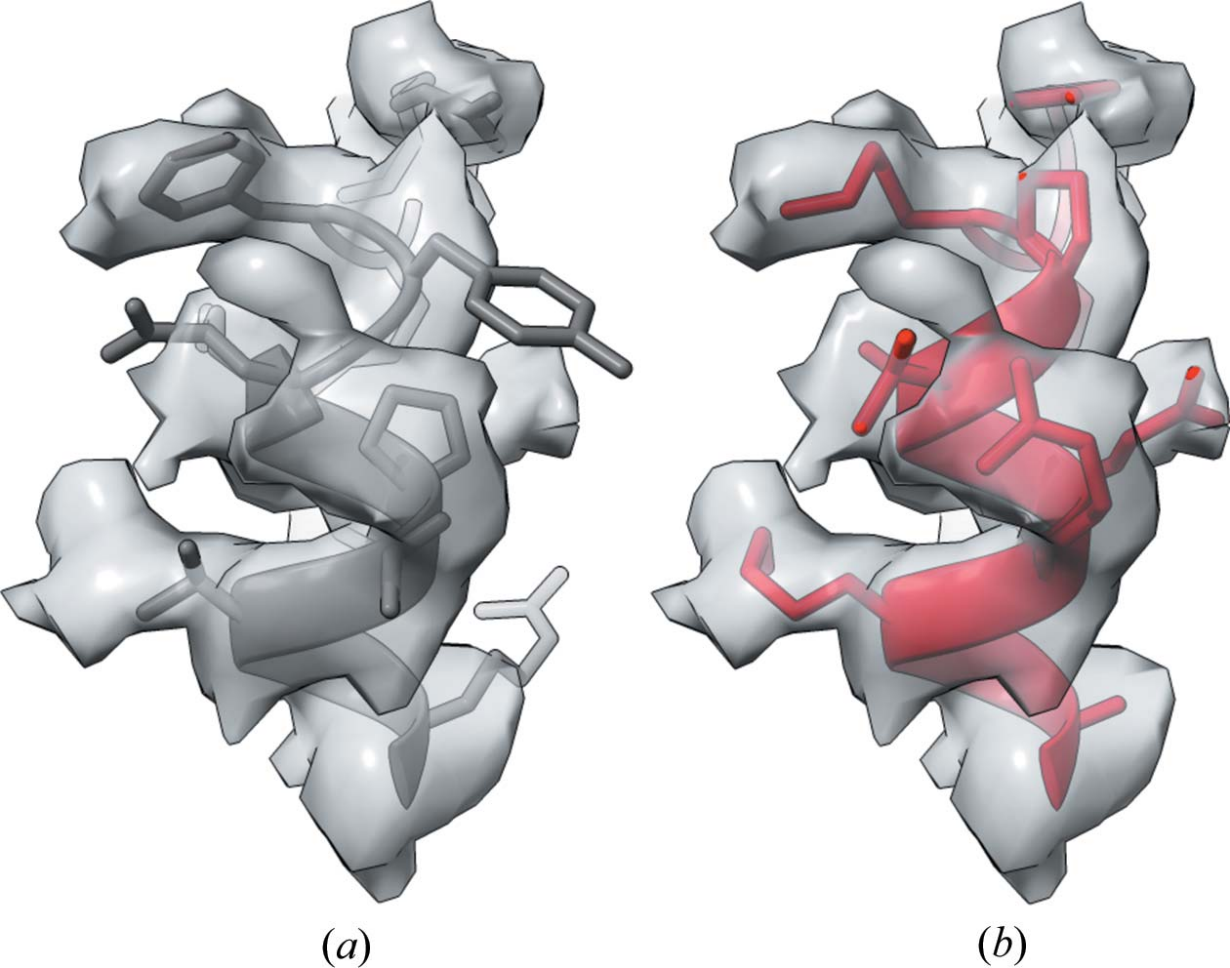
Fragment of an S21 protein model from *E. coli* 70S ribosome at 3.0 Å resolution (PDB ID/EMDB ID 5we4/8814). (*a*) In the deposited coordinates, many side chains outside a well resolved map and a proline inside a regular alpha helix may raise suspicion. (*b*) After sequence re-assignment and side-chain rebuilding with *findMy­Sequence*, the map features are better explained by the model. Only residue range 34–44 in chain u and a corresponding map are shown for clarity.

In the sequence-assignment results we encountered three clear outliers with *p* value below 1 × 10^−4^  and assigned sequences not matching the reference model (protein S21 in models of *E. coli* 70S ribosome at 3.0 Å resolution; PDB IDs/EMDB IDs 5we4/8814, 5wfs/8829 and 5wdt/8813) (Fislage *et al.*, 2018 ▸). A closer look revealed that the reference models had obvious sequence register errors and therefore were removed for the benchmark set (Fig. 5 ▸). Interestingly, all three models were built based on an earlier model of *E. coli* 70S ribosome (PDB ID/EMDB ID 5afi/2849 at 2.9 Å, not in our benchmark set) (Fischer *et al.*, 2015 ▸; Bharat *et al.*, 2015 ▸) that also contains a register error in the corresponding chain.

### 3.4. Crystallography of proteins from natural sources

We collected diffraction datasets from crystal of a protein purified from *B. atrox* that was observed to have phospho­lipase A2 (PLA2) activity. *SIMBAD*’s *MoRDa* database search was performed using default parameters and a model database was created on 18 September 2019.

**Figure 6:**
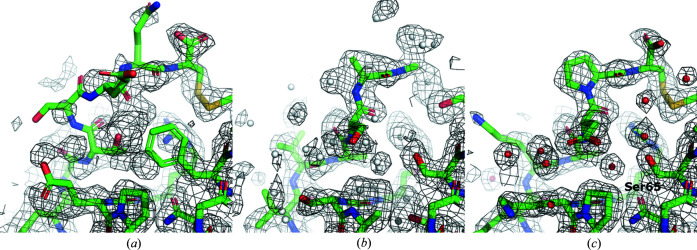
Consecutive steps of crystal structure determination and sequence identification of a protein with hemolytic activity purified from *B. atrox* venom. (*a*) Initial MR solution after 30 *REFMAC5* refinement cycles with jelly-body restraints. The same fragment in (*b*) the *ARP/wARP* model re-traced without an input sequence and used as an input for *findMy­Sequence*, and in (*c*) the final model. The 2*F*_o_ − *F*_c_ maps are contoured at the 1σ level above the mean. The free atoms used for sparse electron-density map representation in *ARP/wARP* are shown as grey spheres. Water molecules are shown as red spheres.

*SIMBAD* identified, as a search model, a PLA2 homologue purified from *Deinagkistrodon acutus* (PDB ID 1mc2; Liu *et al.*, 2003 ▸), which agreed with the protein activity observed. *Phaser* (McCoy *et al.*, 2007 ▸) placed one copy of the search model in the ASU with a log-likelihood gain of 855 and a translation-function *Z* score of 23.6. The model refined to an *R*/*R*_free_ of 0.31/0.33 after 30 cycles of *REFMAC5* (Murshudov *et al.*, 2011 ▸) refinement with jelly-body restraints. The moderate *R*/*R*_free_ factor values after refinement were related to differences between the search model and the target that were clearly visible in the map [Fig. 6 ▸(*a*)]. An automated model rebuilding using *ARP/wARP* without an input sequence improved main-chain traces in many regions [Fig. 6 ▸(*b*)]. For the sequence identification we used a venom proteome determined previously for five specimens of *B. atrox* snakes from two distinct Brazilian Amazon rainforest populations (Amazonas *et al.*, 2018 ▸). Using *findMy­Sequence* and an initial *ARP/wARP* model built without an input sequence [Fig. 6 ▸(*b*)] we identified all six known PLA2 sequences in the venom proteome. The *hmmsearch* *E* values for the hits clearly correlate with the sequence similarity of the sequences to the top-scored sequence variant (Table 1 ▸). A final model was built for the top-scored sequence (A0A1L8D5Z7) that has 68% identity to the MR search model used to solve the structure. The crystal structure model was initially traced using *ARP/wARP*, rebuilt manually in *Coot* and refined in *REFMAC5* version 5.8.0267 using the *CCP4 Cloud* interface (Krissinel *et al.*, 2018 ▸). The model was refined to an *R*/*R*_free_ of 0.19/0.23 [Fig. 6 ▸(*c*)]. The data-collection and structure-refinement statistics are summarized in Table 2 ▸.

In the final refined model we were able to identify a number of amino acids that unambiguously exclude the PLA2 sequence variants other than the top-scored A0A1L8D5Z7 sequence. These include a clearly resolved Ser65 [Fig. 6 ▸(*c*)], which in all other sequence variants is replaced with a tyrosine or phenyl­alanine. A careful manual inspection of the final model did not reveal any possible sequence mismatches. We also queried the venom proteome using *phmmer* from the *HMMER* suite and the sequence of the MR search model identified by *SIMBAD*. The query identified all six PLA2 sequences, although the order of hits was different than in the *findMy­Sequence* results presented in Table 1 ▸ and the sequence with 86% sequence identity to the top hit was scored best.

### 3.5. Identification of Voa1 assembly factor density in yeast V-ATPase Vo proton channel

**Figure 7:**
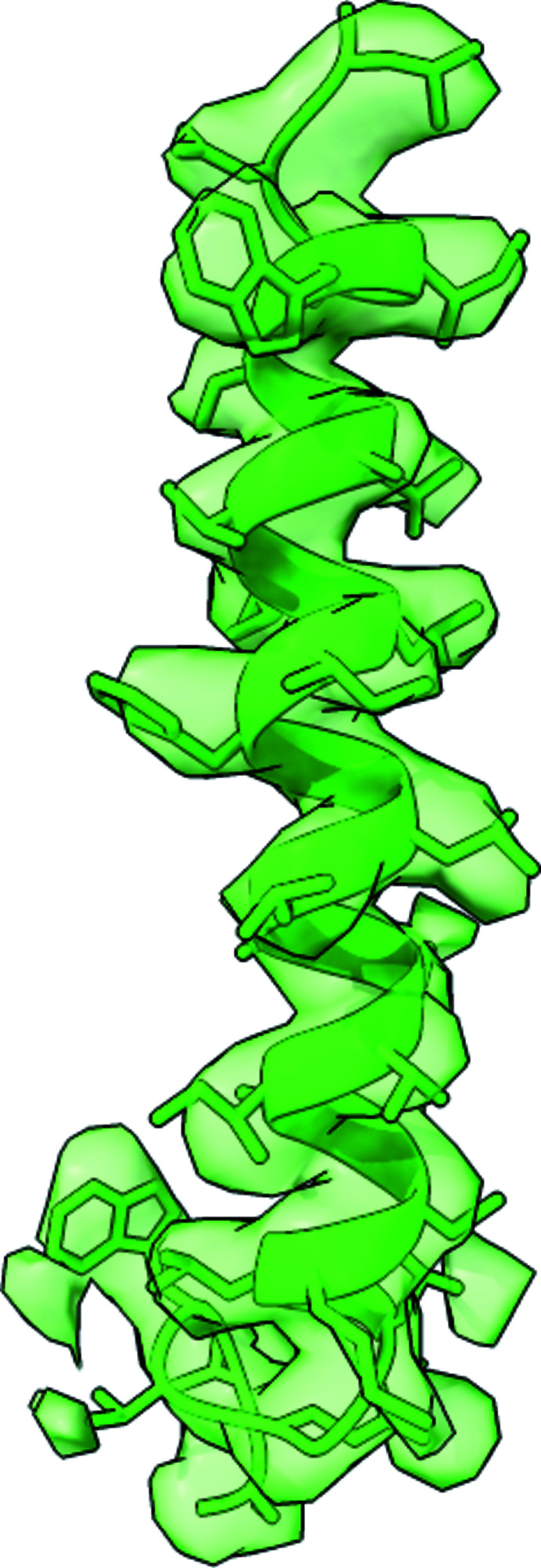
A model of the Voa1 assembly factor and a corresponding cryo-EM reconstruction at 3.5 Å resolution (PDB and EMDB IDs 6c6l and 7348, respectively) (Roh *et al.*, 2018 ▸). Only a residue range of 217–247 is shown for clarity.

A 3.5 Å cryo-EM structure of yeast V-ATPase Vo proton channel revealed an α-helical density (Fig. 7 ▸) inside the central pore of the structure extending into the lumen of the cytoplasm suggesting that it may belong to a separate and unknown protein (Roh *et al.*, 2018 ▸). The authors used mass spectrometry to identify this component as a Voa1 assembly factor. As Voa1 was not required for Vo assembly and deletion of the Voa1 was not lethal, the result could be further confirmed with another EM reconstruction purified from a yeast strain with the Voa1 gene deleted, which was clearly missing the characteristic Voa1 density. We were able to unambiguously identify the Voa1 assembly factor with *findMySequence* in the yeast proteome (*E* value = 1.2 × 10^−13^). We were also able to repeat the result with a single 27 residues long α-helix built into the Voa1 reconstruction using the ‘place helix here’ tool and real-space refinement with secondary structure restraints in *Coot* (an *E* value of 2.4 × 10^−7^ for the correct orientation and no result for a reversed helix). The result could be further confirmed with the sequence-assignment procedure that unambiguously recognized the correct helix orientation and built the model side chains (*p* values of 1.3 × 10^−3^ and 0.9 for correct and reversed helix orientation, respectively). This result confirms the ability of the method to correctly discriminate wrongly traced main-chain fragments.

### 3.6. Discussion and conclusions

We have presented a complete pipeline for the identification of unknown proteins in crystallography and cryo-EM. A key element in the pipeline is a computer program *findMySequence* that identifies the most plausible protein sequence in a sequence database, given an electron-density map or cryo-EM reconstruction and a main-chain-only model.

We have shown that our approach can successfully identify proteins, based on non-curated models automatically built into cryo-EM maps, at local resolutions up to 4.5 Å where models are usually highly fragmented and prone to tracing errors. Indeed, in an earlier study it was shown that the correctness of models built into cryo-EM maps using *ARP/wARP* (*i.e.* the fraction of an automatically built model backbone that is correct) is over 90% up to 4.0 Å local resolution and clearly reduces at lower local resolutions (Chojnowski *et al.*, 2021 ▸). In the same work it was also shown that the dependence of model coordinate errors on local resolution is relatively weak, in particular when they are not assigned to the target sequence. This suggests that the model correctness may affect the sequence-prediction accuracy far more strongly than the accuracy of atomic positions. We also showed that the method’s performance increases for deposited coordinates for which successful sequence identification is possible up to ∼5.5 Å local resolution. In a more realistic experimental setup, one can expect that the method performance will be comparable with this limit, as many errors in automatically traced cryo-EM models can usually be easily corrected manually (Lawson *et al.*, 2021 ▸). We also expect that the use of model-building software other than *ARP/wARP* [*e.g.* *phenix.map_to_model* (Terwilliger, Adams *et al.*, 2018 ▸), *Buccaneer* (Hoh *et al.*, 2020 ▸), *MAINMAST* (Terashi & Kihara, 2018 ▸), *Rosetta* (Wang *et al.*, 2016 ▸) or *DeepTracer* (Pfab *et al.*, 2021 ▸)] should produce comparable results. It must be stressed, however, that most of the available programs require the target sequence on input, and building a model before it becomes available may require non-standard input configuration *e.g.* the use of poly-Ala sequences. To address this issue we also showed that alpha helices, which are relatively easy to build in medium-resolution cryo-EM maps, are a good basis for the sequence-identification procedure.

We observed that in cryo-EM structures our approach often identifies sequences that are very close but not identical to the target. In crystallography, a sequence assignment can be validated after model refinement as residue-type mismatches usually produce elevated *R*-factor values and prominent difference density peaks. In cryo-EM, however, the sequence-assignment validation is much more difficult. Although much like in crystallography they can be detected using difference *F*_o_ − *F*_c_ maps (Yamashita *et al.*, 2021 ▸) and often affect main-chain geometry, to the best of our knowledge, no automated tool exists that could be used for that purpose (Lawson *et al.*, 2021 ▸). Therefore, we proposed a sequence-alignment procedure based on the machine-learning residue-type classifier used in this work. We have shown that it can unambiguously identify the correct sequence assignments of protein chains at a wide range of local resolutions, even though at lower resolutions longer fragments may be needed to produce statistically relevant scores.

The main strength of our approach is not related to the use of potent neural-network classifiers, but to a careful design of the residue-type prediction procedure. As shown by the authors of the *DeepTracer* program, neural-network models are capable of prediction of residue-type probabilities directly in the EM-map voxels. For sequence assignment, this information is combined with independently traced main-chain models. In *findMy­Sequence* we implemented a different approach where input to a residue-type classifier are map points from a region where side-chain moieties are expected for a specific main-chain conformation. This makes the models very sensitive to backbone correctness, enables reliable discrimination of mistraced model fragments and reduces the rate of erroneous sequence assignments. Indeed, we have shown that our approach can correctly identify sequences of automatically traced error-prone protein models. This functionality of *findMy­Sequence* has also proved crucial in building an atomic model of the mycobacterial ESX-5 type VII secretion system into a 3.4 Å resolution cryo-EM map (Beckham *et al.*, 2021 ▸).

To further illustrate the practical use of *findMy­Sequence* we described the identification of a sequence of a protein purified and crystallized from the venom of a *B. atrox* species snake. Prior to the crystallization, only the biochemical activity of the protein was known. Nevertheless, we were able to solve the structures using the brute-force MR approach implemented in *SIMBAD* and then identify the sequence using *findMy­Sequence* with minimal manual input.

For training the *findMy­Sequence* residue-type classifiers we used deposited cryo-EM maps and automatically refined PDB models, ignoring all side chains. However, map sharpening and the use of available cryo-EM map modification approaches (*e.g.* Jakobi *et al.*, 2017 ▸; Terwilliger, Sobolev *et al.*, 2018 ▸; Ramírez-Aportela *et al.*, 2020 ▸) may improve map interpretability and thereby increase initial model completeness, which is one of the most important factors affecting the sequence-identification-procedure reliability. Similarly, placement of some predicted side-chain moieties in the models may improve the quality of their main-chain geometry and hence lead to better sequence-identification results.

Although benchmark results that are presented this work are based on monomeric structures, our approach can be successfully applied to multimers. This, however, requires a sequence identification based on manually selected intermediate model fragments in an iterative model-building procedure. Furthermore, we would like to stress here that *findMy­Sequence* is very fast and can be conveniently used during manual model building. In our benchmarks, a single sequence-identification procedure took less than 30 s for models containing up to several thousand residues. This is negligible compared with the time usually required to complete an automated model-building procedure, which, for example, in *ARP/wARP* averages 12 h for cryo-EM models (Chojnowski *et al.*, 2021 ▸).

Our results show that availability of an initial model is a main limitation of the method application in crystallography. In our crystal structure benchmark set every promising MR solution, with *R*_free_ below 50%, could be used for the successful identification of target sequences. This required a search model with at least 20% sequence identity to the target, which agrees with a popular rule of thumb for search-model suitability (Abergel, 2013 ▸). Thus, where an MR search is likely to be successful, the resulting map is likely to be tractable for *findMy­Sequence*. Although at lower resolutions this does not always guarantee success, one may expect that, owing to the number of available protein structures, brute-force MR will often be able to provide an initial model for sequence identification (Simpkin *et al.*, 2018 ▸). For benchmarks we used three good-quality diffraction datasets, with data resolution being the major limiting factor. Crystallographers, however, often work with datasets bearing a number of pathologies, like twinning or severe anisotropy. These, no doubt, would affect the performance of the approach and may require more effort from a crystallographer *e.g.* determination of experimental phases or manual model building. Nevertheless, the application of our method should save significant amounts of a structural biologist’s time in the majority of cases.

## 4. Related literature

The following reference is cited in the supporting information for this article: Larkin *et al.* (2007 ▸).

## Supplementary Material

Supporting figures. DOI: 10.1107/S2052252521011088/pw5018sup1.pdf

PDB reference: Crystal structure of PLA2 from snake venom of Peruvian *Bothrops atrox*, 7m6c

## Figures and Tables

**Table 1:** Results of the sequence identification in *B. atrox* venom proteome using *findMy­Sequence* and an *ARP/wARP* PLA2 model built without an input sequence

Uniprot ID	*hmmsearch* *E* value	Sequence identity to the top hit (%)
A0A1L8D5Z7	1.40 × 10^−28^	100
A0A1L8D611	2.10 × 10^−23^	86
A0A1L8D605	1.00 × 10^−17^	61
A0A1L8D602	9.30 × 10^−16^	56
A0A1L8D6C4	3.00 × 10^−15^	52
A0A1L8D5Z4	4.10 × 10^−12^	50

**Table 2:** Data-collection and structure-refinement parameters for PLA2 from *B. atrox* Lowest-resolution-shell values are given in brackets.

Data collection	
Space group	*P*22_1_2_1_
*a*, *b*, *c* (Å)	36.40, 58.36, 64.46
α, β, γ (°)	90, 90, 90
Resolution (Å)	43.26–1.95 (2.06–1.95)
Completeness (%)	99.5 (99.9)
Wavelength (Å)	1.46
Multiplicity	4.0 (4.1)
〈*I*/σ(*I*)〉	5.8 (2.2)
CC_1/2_ (%)	99.2 (83.8)
*R*_merge_ (%)	9.9 (32.2)
	
Refinement statistics	
*R*_work_/*R*_free_ (%)	19/23
*B* factors (Å^2^)	
Wilson	23
Model average	27
Protein	21
Solvent	32.1
R.m.s. deviations	
Bond lengths (Å)	0.01
Bond angles (°)	1.92
Ramachandran plot (%)	
Favoured	95.9
Allowed	3.3
Outliers	0.8
*MolProbity* clashscore	4
