# Supplementary material for: findMySequence: a neural-network-based approach for identification of unknown proteins in X-ray crystallography and cryo-EM
Source: IUCrJ. 2021 Dec 1;9(Pt 1):86–97. doi: 10.1107/S2052252521011088 (PMC8733886; doi:10.1107/S2052252521011088)
Supplement: Supplementary file 1 [file m-09-00086-sup1.pdf]

IUCrJ

Volume 9 (2022)

Supporting information for article:

findMySequence: a neural-network-based approach for identification of unknown proteins in X-ray crystallography and cryo-EM

Grzegorz Chojnowski, Adam J. Simpkin, Diego A. Leonardo, Wolfram Seifert-Davila, Dan E. Vivas-Ruiz, Ronan M. Keegan and Daniel J. Rigden

Supplement

findMySequence: a neural-network-based approach for identification of unknown proteins in X-ray crystallography and cryo-EM

Grzegorz Chojnowski[1,#], Adam J. Simpkin[2], Diego A. Leonardo[3], Wolfram Seifert-Davila[4], Dan E. Vivas-Ruiz[5], Ronan M. Keegan[6], Daniel J. Rigden[2]

[1]European Molecular Biology Laboratory, Hamburg Unit, Notkestrasse 85, 22607 Hamburg, Germany
[2]Institute of Systems, Molecular and Integrative Biology, University of Liverpool, Liverpool L69 7ZB, England
[3]São Carlos Institute of Physics, University of São Paulo, Avenida João Dagnone 1100, São Carlos, SP 13563-120, Brazil
[4]European Molecular biology Laboratory. Meyerhofstraße 1, 69117, Heidelberg, Germany
[5]Laboratorio de Biolgía Molecular, Facultad de Ciencias Biológicas, Universidad Nacional Mayor de San Marcos, Av. Venezuela Cdra 34 S/N, Ciudad Universitaria, Lima, Peru
[6]UKRI-STFC, Rutherford Appleton Laboratory, Research Complex at Harwell, Didcot OX11 0FA, England

# Correspondence: gchojnowski@embl-hamburg.de

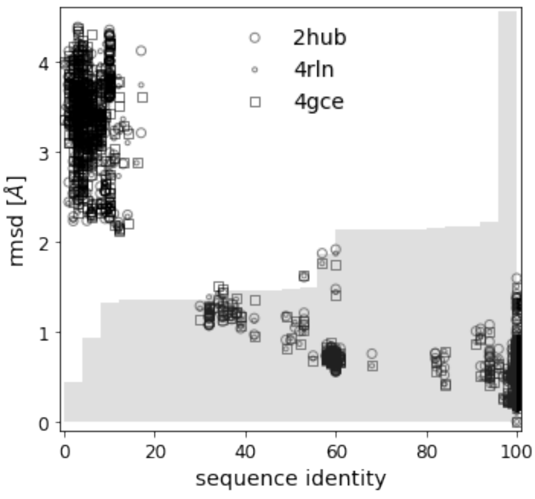

Figure S1 Dependence between CA-atom positions root mean square difference (rmsd) and sequence identity for the hen egg-white lysozyme crystal structure identification benchmark set target structures (PDB id codes 2hub, 4rln, and 4gce) and corresponding search models. Sequence identities and rmsd values were calculated using CLUSTALW2 (Larkin *et al.*, 2007) and GESAMT (Krissinel, 2012) respectively with default parameters. A cumulative histogram of the benchmark set search models to corresponding targets is shown in grey (in arbitrary units).

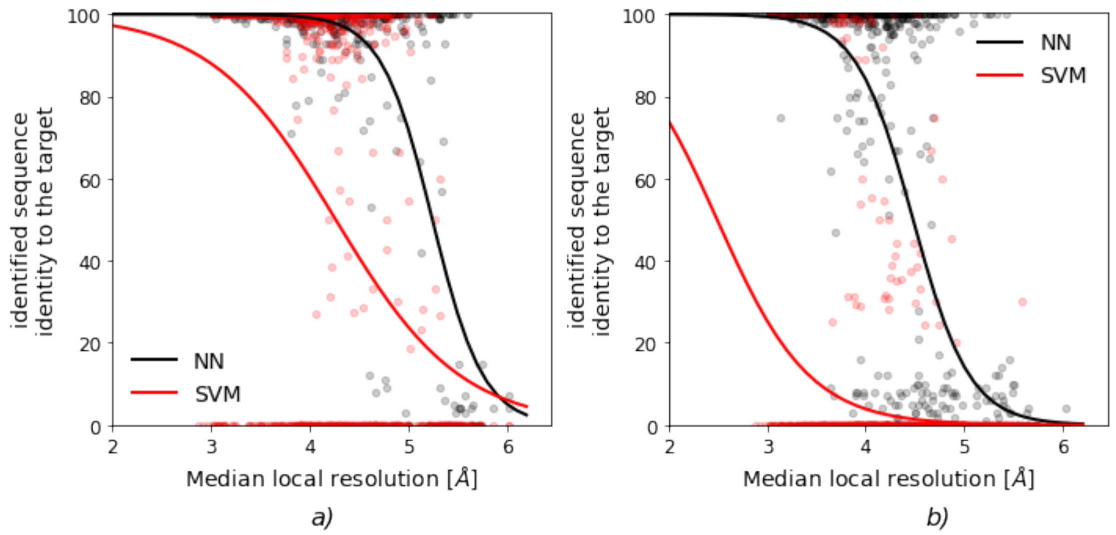

Figure S2 Comparison of sequence identification benchmark results for 909 cryo-EM models of ribosomal proteins and corresponding proteomes for a Neural Network (NN) residue type classifier presented in this work and corresponding Support-Vector Machine (SVM) classifier implemented in ARP/wARP 8.0 (Chojnowski *et al.*, 2019). The two methods were compared for a) refined deposited coordinates and b) models built *de novo*. The continuous curves are logistic-regression estimates of a probability that an identified sequence will have at least 80% sequence identity to the target sequence.

References

Chojnowski, G., Pereira, J. & Lamzin, V. S. (2019). *Acta Crystallogr. Sect. D Struct. Biol.* **75**, 753–763.

Krissinel, E. (2012). *J. Mol. Biochem.* **1**, 76.

Larkin, M. A., Blackshields, G., Brown, N. P., Chenna, R., McGettigan, P. A., McWilliam, H., Valentin, F., Wallace, I. M., Wilm, A., Lopez, R. & others (2007). *Bioinformatics*. **23**, 2947–2948.
